# Regulation of Mitochondrial Homeostasis by sAC-Derived cAMP Pool: Basic and Translational Aspects

**DOI:** 10.3390/cells10020473

**Published:** 2021-02-22

**Authors:** Muhammad Aslam, Yury Ladilov

**Affiliations:** 1 Experimental Cardiology, Department of Internal Medicine I, Justus Liebig University, Aulweg 129, 35392 Giessen, Germany; muhammad.aslam@physiologie.med.uni-giessen.de; 2 Department of Cardiology, Kerckhoff Clinic GmbH, 61231 Bad Nauheim, Germany; 3 DZHK (German Centre for Cardiovascular Research), Partner Site Rhein-Main, 61231 Bad Nauheim, Germany; 4 Independent Researcher, 22393 Hamburg, Germany

**Keywords:** soluble adenylyl cyclase, cAMP, PKA, EPAC, OXPHOS, mitochondrial biogenesis, mitophagy, apoptosis, mitochondrial dynamics

## Abstract

In contrast to the traditional view of mitochondria being solely a source of cellular energy, e.g., the “powerhouse” of the cell, mitochondria are now known to be key regulators of numerous cellular processes. Accordingly, disturbance of mitochondrial homeostasis is a basic mechanism in several pathologies. Emerging data demonstrate that 3′–5′-cyclic adenosine monophosphate (cAMP) signalling plays a key role in mitochondrial biology and homeostasis. Mitochondria are equipped with an endogenous cAMP synthesis system involving soluble adenylyl cyclase (sAC), which localizes in the mitochondrial matrix and regulates mitochondrial function. Furthermore, sAC localized at the outer mitochondrial membrane contributes significantly to mitochondrial biology. Disturbance of the sAC-dependent cAMP pools within mitochondria leads to mitochondrial dysfunction and pathology. In this review, we discuss the available data concerning the role of sAC in regulating mitochondrial biology in relation to diseases.

## 1. Introduction

3′–5′-Cyclic adenosine monophosphate (cAMP) is a ubiquitous and essential intracellular second messenger molecule involved in a plethora of physiological processes. cAMP signalling, even though discovered more than half a century ago, remains an object of basic and translational research. Generally, cAMP signalling consists of three components: (i) enzymes synthesizing cAMP, i.e., adenylyl cyclases; (ii) enzymes degrading cAMP, i.e., phosphodiesterases (PDEs); and (iii) effectors such as protein kinase A (PKA), exchange protein directly activated by cAMP (EPAC), and cyclic nucleotide-gated ion channels. PKA and EPAC are among the major known mediators of cAMP signalling. PKA is serine/threonine kinase that consists of two regulatory and two catalytic subunits forming an inactive holoenzyme complex [1]. Upon binding of cAMP to the regulatory complex, the constitutive active catalytic subunit is released, which phosphorylates its substrate [2]. EPAC is a guanine nucleotide exchange factor (GEF) for small GTPases Rap1 and 2 (members of Ras family of GTPases) [3] and is involved in a variety of cellular processes such as cell proliferation and differentiation, actin cytoskeleton dynamics, cell adhesion and polarity, and exocytosis [4]. There are two known isoforms of EPAC, i.e., EPAC1 and EPAC2, the expression of which is cell type-specific and shows distinct activation patterns in response to cAMP-raising stimuli [5]. There are two main sources of cAMP in the cell: transmembrane (tmAC) and intracellularly localized soluble adenylyl cyclases (sAC). In mammalian cells, nine genes (Adcy1-9) encode tmACs and one gene (Adcy10) encodes sAC. Two important properties characterize the principal difference between tmAC and sAC: first, G-proteins regulate tmAC activity, whereas sAC activity is stimulated by bicarbonate and Ca^2+^ [6] as well as by its substrate adenosine 5´-triphospahte (ATP) [7]. Second, the localization of tmAC is restricted to the plasma membrane, whereas sAC is widely distributed within the cell and inside organelles [8].

The distinct spatial distribution of the two main cAMP sources leads to the formation of multiple intracellular cAMP compartments, thereby enabling the specificity and selectivity of cAMP signalling. The compartmentalization of cAMP signalling within the cell is achieved through the restriction of cAMP diffusion by cAMP degrading PDEs [9,10] and scaffolding proteins such as A-kinase anchoring proteins (AKAPs). AKAPs are localized at specific subcellular sites and play a key role in maintaining subcellular compartmentalization of cAMP signalling by generating spatially discrete signalling complexes that create local gradients of cAMP and/or cAMP effectors [11,12,13]. With the exception of a rare internalization of tmAC [14], the cAMP pool produced by tmAC under physiological conditions is mainly localized close to the plasma membrane. In contrast, sAC forms cAMP pools within various cellular compartments, e.g., the cytosol, mitochondria, the nucleus, or the subplasmalemmal compartment [15,16,17,18]. Such distinct subcellular distribution allows specific regulation of various cellular functions such as cell death and growth [19], migration [18], gene expression [20], and ATP synthesis [21]. Consequently, dysregulation of sAC-derived cAMP pools may lead to various pathologies. A detailed review on the functional significance of the sAC-generated cAMP pools can be found in [22]. In the present review, we focus on the role of sAC-dependent cAMP signalling in regulating mitochondrial homeostasis. In particular, we discuss the impact of the dysregulation of sAC signalling on mitochondrial biology and its relation to various diseases.

## 2. Role of the sAC-Signalling in Mitochondrial Biology

In mammalian cells, the inner mitochondrial membrane is impermeable to cAMP under physiological conditions [10,11,17]. Several reports demonstrated that mitochondrial sAC, localized in the mitochondrial matrix, controls the intra-mitochondrial cAMP pool independently of cytosolic cAMP signalling [10,17,18]. PDE2 was shown to be the main cAMP-degrading PDE isoform in the mitochondrial matrix [23]. In addition, there are two other mitochondrial cAMP pools that may also be controlled by sAC: the intermembrane space (IMS) and the outer mitochondrial membrane (OMM) [24,25].

PKA and EPAC, two important cAMP effectors, are also present in the mitochondrial matrix, the IMS, and the OMM [24,25]. Several reports suggested there are a large number of PKA phosphorylation targets in mitochondria [26,27]. Similarly, EPAC targets localized in mitochondria have also been reported [28,29].

### 2.1. sAC and OXPHOS Activity

**Figure 1:**
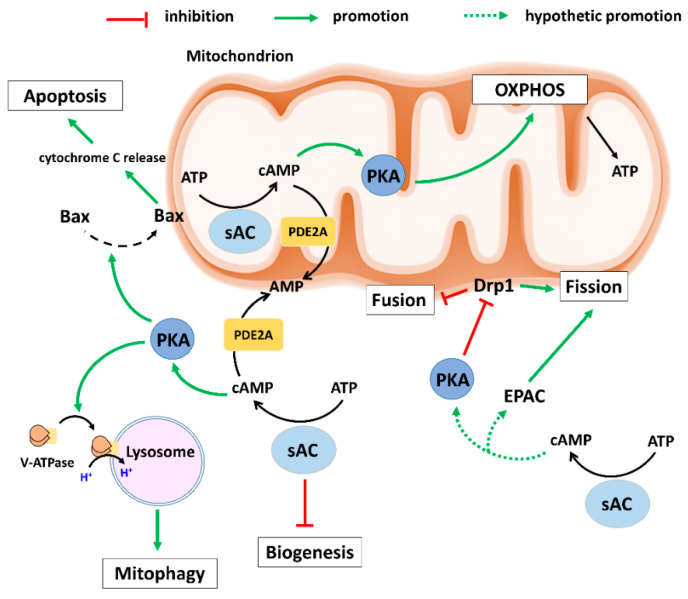
Schematic presentation of the effects of sAC-signalling on mitochondrial biology. Intramitochondrial sAC-signalling activates OXPHOS leading to ATP production. Under various stresses, extramitochondrial sAC–PKA axis promotes the recruitment of pro-apoptotic Bax to mitochondria and induces cytochrome c release leading to apoptosis. Furthermore, extramitochondrial sAC–PKA axis supports the removal of defective mitochondria (Mitophagy) by inducing recruitment of V-ATPase to lysosomes. Moreover, extramitochondrial sAC-signalling inhibits mitochondrial biogenesis via a still unknown mechanism. The extramitochondrial PKA and EPAC regulate mitochondrial dynamics and may be activated by the sAC-derived cAMP pool. AMP: Adenosine monophosphate; ATP: Adenosine 5′-triphosphate; cAMP: cyclic AMP; EPAC: Exchange protein directly activated by cAMP; Drp1: Dynamin-related protein 1; OXPHOS: oxidative phosphorylation; PDE2A: Phosphodiesterase 2A; PKA: Protein kinase A; sAC: soluble adenylyl cyclase; V-ATPase: Vacuolar-ATPase.

A seminal study by Acin-Perez [15] discovered the presence of a sAC–PKA signalling axis in the mitochondrial matrix and its role in regulating oxidative phosphorylation (OXPHOS). Generally, it has been shown that cAMP produced in the mitochondrial matrix by sAC promotes OXPHOS via PKA-dependent phosphorylation of cytochrome c oxidase subunit IV [15,30]. Deletion of sAC in fibroblasts disturbed OXPHOS, whereas restoring sAC expression in the mitochondrial matrix rescues the process [31]. Similar results showing regulation of OXPHOS activity via the cAMP–PKA axis were obtained in yeast, where the inhibition of sAC diminished mitochondrial respiration and OXPHOS activity [32]. Furthermore, in human dermal fibroblasts, De Rasmo et al. [33] found that inhibition of sAC reduced complex I activity, which was rescued by adding a membrane-permeable cAMP analogue. Interestingly, the authors showed that intramitochondrial cAMP exerts this positive effect on complex I by preventing degradation of nuclear-encoded subunits of complex I by mitochondrial proteases, whose activity is promoted by inhibition of sAC or PKA. Later, in a follow-up study, the same group showed that sAC-generated cAMP regulates the ATP hydrolytic and synthase activities as well as stability of some stalk subunits of FoF_1_ ATP synthase [34]. Therefore, the intramitochondrial sAC–PKA axis may support OXPHOS activity via (i) direct PKA-dependent phosphorylation and/or (ii) via reducing degradation of OXPHOS proteins (Figure 1).

Emerging data suggest several mechanisms regulating mitochondrial respiration and ATP synthesis via an sAC-dependent pathway. In particular, direct stimulation of sAC by bicarbonate or Ca^2+^ promotes elevation of the cAMP concentration in the mitochondrial matrix and, as a result, activates OXPHOS activity [22,35]. A recent report by Hebert-Chatelain et al. [36] also suggested that the activation of mitochondria-localized type-1 cannabinoid receptors (mtCB1) leads to sAC inhibition accompanied by decreased mitochondrial cAMP concentration, complex I activity, mitochondrial respiration, and cellular ATP content in cultured neuronal cells. The study showed the importance of the PKA-dependent phosphorylation of complex I subunit NDUFS2 in mitochondrial function.

Aside from sAC, intramitochondrial cAMP signalling is also regulated by PDEs. PDE2A is the most predominant intramitochondrial isoform [23]. Importantly, PDE2 is activated by cGMP, which enables a negative cGMP–cAMP cross-talk within mitochondria [23,37]. PDE2A is also localized outside of the mitochondrial matrix, in particular at the outer or inner mitochondrial membrane where it regulates the mitochondrial membrane potential, mitochondrial morphology, and cell death [38]. The activation of PDE2A by cGMP points to a potential regulation of its activity by NO signalling. Indeed, our recent report revealed that activation of NO signalling in cardiac cells by estradiol reduced mitochondrial cAMP concentration in a soluble guanylyl cyclase (sGC)- and PDE2A-dependent manner [16]. Furthermore, the localization of sGC in mitochondria was confirmed by western blot analysis in this study. Activation of the mitochondrial sGC-PDE2A axis followed by decreased mitochondrial cAMP levels resulted in a significant reduction in mitochondrial cytochrome c oxidase activity [16]. These data agree with a previous report that applied a selective PDE2A inhibitor, BAY60-7550, that resulted in an increased oxygen consumption rate and ATP production in isolated mitochondria [23]. The sAC/cAMP signalling effects on mitochondrial biology are schematically presented in Figure 1.

### 2.2. sAC and Mitochondrial Biogenesis

Mitochondrial biogenesis, first described by Holloszy in 1967 [39], is the process by which the cells increase their mitochondrial content by increasing the growth and division (fission) of pre-existing mitochondria. Accumulating data suggest cAMP signalling positively modulates mitochondrial biogenesis in a variety of mammalian cell types [40,41,42] and in yeast [43], emphasizing the evolutionarily conserved role of cAMP signalling. Pharmacological activation of tmAC in human adipocytes increased mitochondrial copy number and DNA content [44]. Likewise, pentoxyfilline, a non-selective PDE inhibitor, increased cellular cAMP content and induced mitochondrial biogenesis in aged rat brains, thereby alleviating motor and cognitive deficits [45].

cAMP mediates its effects on mitochondrial biogenesis via activation of downstream effectors, including PKA and EPAC. Indeed, immobilization stress in rats induces mitochondrial biogenesis in Leydig cells in vivo, which is accompanied by an upregulation of cAMP levels and enhanced PKA activity [46]. The authors showed that cAMP signalling stimulates expression of peroxisome proliferator-activated receptor gamma co-activator 1 alpha (PGC1α) through a mechanism involving PKA. Similarly, the pharmacological elevation of the cellular cAMP concentration by resveratrol, a polyphenol in red wine, mediated via inhibition of PDE4, increases PGC1α activity (as a result of the PGC1α deacetylation) and mitochondrial biogenesis in mouse C2C12 myotubes [47]. The authors suggest EPAC as a key mediator of the effects of resveratrol, which leads to the activation of 5′ AMP-activated protein kinase (AMPK) and Sirt1. Furthermore, PGC1 alpha and several mitochondrial genes such as ND2, ND4, and ND5 have CREB binding sequences and, therefore, can be activated by cAMP signalling via PKA-dependent CREB phosphorylation [48]. Although CREB is known to be a nuclear transcription factor, it is also localized inside mitochondria [49]. Depletion of mitochondrial CREB results in suppression of several mitochondrial-encoded genes of complex I accompanied by reduced complex I activity [48].

On the other hand, dysregulation of cAMP signalling is one of the main mechanisms of mitochondrial dysfunction and disturbed mitochondrial biogenesis. For example, in unilateral ureteral obstruction-induced renal fibrosis, the cAMP degrading enzyme PDE4 is upregulated, which leads to reduced cellular cAMP levels and mitochondrial biogenesis. Inhibition of PDE4 normalizes mitochondrial function and biogenesis via cAMP/EPAC signalling [42].

Altogether, these studies demonstrate a key role of cAMP signalling in mitochondrial biogenesis. However, little is known about the role of the sAC-generated cAMP pool. sAC knockout cells totally lacking sAC exhibit defective OXPHOS but upregulated mitochondrial biogenesis and high levels of total mitochondrial content, suggesting a compensatory mechanism is activated to support the reduced OXPHOS activity in these cells [31]. Overexpressing the total or mitochondrial sAC in this study corrected the OXPHOS defects and normalized the compensatory mitochondrial biogenesis. Interestingly, a study by Acin-Perez et al. [21] showed that that transient sAC expression in 293T HEK cells significantly reduced mitochondrial biogenesis. These studies suggest that under physiological or pathological conditions, sAC acts as a negative regulator of mitochondrial biogenesis. For schematic presentation please see Figure 1.

### 2.3. sAC and Mitochondrial Dynamics

In order to maintain their shape, size, and localization, mitochondria are constantly undergoing coordinated cycles of fusion and fission, known as mitochondrial dynamics. These processes are regulated by dynamin-like GTPases and are crucial for several cellular processes such as the cell cycle, differentiation, and apoptosis. Mitofusin 1/2 (Mfn1/2) and optic atrophy 1 (Opa1) mediates mitochondrial fusion, whereas dynamin-related protein 1 (Drp1) mediates mitochondrial fission. Disorders of mitochondrial dynamics are implicated in several human endocrine, neoplastic, neurodegenerative, and cardiovascular disorders [50]. The activities of these regulators of mitochondrial dynamics are influenced by various cellular signalling mechanisms, in particular by cAMP signalling [38,51]. cAMP-mediated activation of PKA at the OMM induces phosphorylation of Drp1 at Ser637, which leads to its inactivation and suppression of mitochondrial fission. This results in mitochondrial elongation and enhanced resistance to apoptotic signals. In contrast, calcineurin dephosphorylates Drp1 and restores its activity, leading to mitochondrial fragmentation [52]. Moreover, PKA phosphorylates Mfn2, leading to mitochondrial elongation and cell growth arrest [53]. In contrast, EPAC activation has been reported to promote mitochondrial fission and its inhibition suppresses mitochondria fission [54], although the mechanism is still unknown.

Aside from its presence at the OMM, PKA appears to be tethered to the IMM via sphingosine kinase type 1-interacting protein (SKIP) [55,56], where it mediates phosphorylation of Coiled-Coil-Helix-Coiled-Coil-Helix Domain Containing 3 (ChChd3), a scaffold protein that is a component of the mitochondrial contact site and cristae organising system [57]. Phosphorylation of ChChd3 by PKA requires SKIP, suggesting that mitochondrial SKIP/PKA complexes have relevant physiological roles in cristae integrity/remodelling and mitochondrial activities [58]. Interestingly, depletion of ChChd3 results in extensive mitochondrial fragmentation [59].

Although the role of cAMP/PKA in controlling mitochondrial dynamics is well established, a direct role of sAC in the context of mitochondrial fission and fusion has not yet been documented. However, based on the data from several studies demonstrating a cAMP pool at the OMM generated by the activity of sAC [60], one may assume that sAC is involved in the regulation of mitochondrial dynamics (Figure 1).

### 2.4. sAC and Mitophagy

The clearance of damaged macromolecules and organelles by selective autophagy is an essential mechanism supporting cell health. Mitophagy, leading to the clearance of dysfunctional mitochondria, is one of the forms of selective autophagy that is crucial for mitochondrial quality control and thus for the maintenance of cell viability [61]. Indeed, accumulation of dysfunctional mitochondria disturbs cellular energy homeostasis and leads to the uncontrolled formation of reactive oxygen species (ROS) and cell death [61]. In mammalian cells, the PTEN-induced putative kinase 1 (PINK1) and the E3 ubiquitin ligase Parkin act in a coordinated fashion to regulate mitophagy. Under normal physiological conditions, PINK1 is continuously imported from the OMM to the IMM where it is cleaved by the protease PARL located in the IMS and exported back to the cytoplasm for degradation [62]. When the mitochondrial membrane potential drops, PINK1 escapes from import to the IMM and cleavage, thus accumulating at the OMM where, via interaction with MIC60, it mediates the recruitment of Parkin and signals for mitophagy. In addition, pro-fission proteins such as Drp1 and autophagy receptors are recruited to complete the fragmentation and elimination of the targeted mitochondrion [24].

Several studies have demonstrated the role of cAMP signalling in regulating mitophagy [24]. In particular, PKA-dependent phosphorylation of Drp1 shifts the balance from fission to fusion, which in turn may disturb the mitophagy process [63]. Accordingly, overexpression of mitochondrial PDE2A results in reduced cAMP levels at the OMM, mitochondrial fragmentation, enhanced recruitment of Parkin at the OMM, and mitophagy [64]. Conversely, pharmacological inhibition of PDE2A results in enhanced PKA activity at the OMM, elongated mitochondria, suppression of Parkin recruitment, and reduced basal mitochondrial clearance [64]. Similarly, PKA-mediated phosphorylation of MIC60 interrupts assembly of the PINK1 complex, resulting in the destabilization of PINK1 on mitochondria. As a result, translocation of Parkin to depolarized mitochondria is prevented and mitochondrial clearance is blocked [65]. Whether sAC may contribute to the regulation of mitophagy remain largely unknown.

Our recent report [66] showed a direct link between sAC and mitophagy in cardiac and endothelial cells. Particularly, downregulation of sAC led to the disturbed mitochondrial clearance that culminated in oxidative stress and energy imbalance. Though the causal link between sAC and mitophagy has not been found in the study, our pilot experiments (unpublished data) suggest that sAC downregulation impairs lysosomal acidification leading to disturbed autophagy/mitophagy flow. In line with these data, a recent report by Rahman et al. [67] revealed the role of sAC in lysosomal acidification due to co-localization of proton-pumping V-ATPase with lysosomes. The authors showed that the deletion of sAC in fibroblasts reduced the degradative activity of lysosomes and led to accumulation of autophagosomes, indicating diminished autophagy flow. Therefore, the activity of sAC seems to be important for autophagy and mitophagy processes under physiological conditions (Figure 1).

### 2.5. sAC and Apoptosis

Our recent studies suggested a role of sAC in apoptotic cell death, particularly in the mitochondrial pathway of apoptosis. In cultured primary neurons [68], cardiomyocytes [69], coronary endothelial cells [70], or smooth muscle cells [71], mitochondrial injury and release of cytochrome C were induced by simulated ischaemia, acidosis, or oxysterols. Mechanistic analysis revealed an essential role of the stress-induced translocation of sAC to the mitochondrial membrane followed by PKA-dependent phosphorylation/activation of the pro-apoptotic Bax protein, its translocation to mitochondria, the release of cytochrome C, and cleavage of caspases-9 and -3. A similar mechanism was reported by another group in human and mouse cholangiocytes challenged with bile salt [72]. In addition, sAC-dependent activation of mitochondrial apoptosis under oxidative stress also involves activation of another pro-apoptotic protein, Bad [73]. Importantly, the sAC-derived cAMP pool involved in mitochondrial apoptosis in our studies is localized outside rather than inside the mitochondria [71] (Figure 1). In contrast, the intra-mitochondrial cAMP pool seems to be protective against apoptosis. Indeed, a recent study by Signorile et al. [74] showed that a decrease in the intra-mitochondrial cAMP concentration causes proteolytic degradation of mitochondrial Sirt3 that, in turn, promotes acetylation and proteolytic processing of OPA1, leading to mitochondrial fission and eventually apoptosis. Therefore, it seems that extra- and intra-mitochondrial sAC-derived cAMP pools have opposite effects on mitochondrial-dependent apoptosis.

## 3. Role of Dysregulation of Mitochondrial sAC Signalling in Pathologies

### 3.1. Ischaemia/Reperfusion

Ischaemia/reperfusion (I/R) injury is a common phenomenon in various surgical settings and chronic cardiovascular and cerebrovascular diseases. Under ischaemic insult, reperfusion is the only way to save cells from irreversible injury. However, reperfusion itself may lead to cell injury in addition to the ischaemic damage. Mitochondria play an essential role in cell survival under I/R stress. Rapid recovery of ATP synthesis during reperfusion is important to restore ATP-consuming cellular processes. In particular, sufficient ATP content is required for rapid extrusion of the excessive cytosolic Ca^2+^ accumulated during ischaemia. This prevents excessive mitochondrial Ca^2+^ uptake leading to the opening of mitochondrial permeability transition pores, ATP depletion, ROS formation, and necrotic cell death [75]. 

As described above (Section 2.1), sAC supports mitochondrial ATP synthesis due to PKA-dependent phosphorylation of certain key proteins in the respiratory chain [15,30]. That sAC may protect cells against I/R injury remained unknown until recently. In a recent investigation, we challenged cardiac cells with simulated I/R and manipulated sAC activity/expression [76]. Inhibition of sAC activity during reperfusion aggravated reperfusion-induced cell death. In contrast, mitochondria-targeted overexpression of sAC preserved cell viability after I/R. Similarly, treatment of cells only during reperfusion with Bay 60-7550, an inhibitor of PDE2, which is the predominant PDE isoform in mitochondria, protected against necrotic cell death. It is worthy to note that this PDE2 inhibition markedly elevated the cAMP concentration in the mitochondrial matrix but not in the cytosol, indicating a mitochondria-specific effect. In agreement with these results, a recent report suggested that PDE2 inhibition has a protective effect in a brain I/R model, although delayed rather than acute effects of reperfusion were analysed [77].

In contrast to our study, a recent report by Fazal et al. [78] suggested that hypoxia-reoxygenation in adult mouse cardiomyocytes leads to cell death and the elevation of cellular cAMP in a sAC-dependent manner. Mechanistic analyses revealed that EPAC1 was activated by sAC and promoted mitochondrial permeability transition pore opening and cell death during hypoxia-reoxygenation. Unfortunately, to demonstrate a causal role of sAC only treatment with the sAC-inhibitor KH7 was used in this study, which has marked non-specific side effects on mitochondria [79]. Therefore, the conclusion drawn from this study regarding the role of sAC should be assessed prudently. Another recent report by Chagtoo et al. [68] demonstrated that sAC promotes I/R injury in rat embryonic neurons. In particular, pharmacological suppression of sAC during reperfusion reduced cellular cAMP levels and ameliorated reperfusion-induced mitochondrial apoptosis and ROS formation. Similarly, sAC knockdown prevented neuronal death. Further analysis revealed a causal role of PKA as a major downstream target of sAC involved in the protection. The underlying mechanism mediating this sAC-dependent apoptosis in neurons, is, most likely, similar to those demonstrated for adult rat cardiomyocytes exposed to simulated I/R [69], i.e., PKA-dependent BAX phosphorylation and translocation to mitochondria. In this scenario, non-mitochondrial sAC contributes to the I/R-induced cell injury.

### 3.2. Preconditioning to I/R Injury

Preconditioning generally refers to adaptation to damaging stress via short pre-treatment with the same stress or other interventions. Recent data suggested the involvement of sAC in preconditioning to I/R injury. In a two-week model of preconditioning with a glucagon-like peptide 1 (GLP-1) metabolite GLP-1(28–36), Siraj et al. [80] demonstrated that GLP-1(28–36) protects against myocardial I/R injury. In their study, pre-treatment with GLP-1(28–36) reduced infarct size, improved cardiac function at the end of reperfusion, and protected coronary vascular cells from oxidative stress. The authors found that the protective effect is mediated through activation of sAC, leading to increased cellular cAMP levels in coronary artery smooth muscle cells and endothelial cells but not in cardiomyocytes. Importantly, cell metabolism experiments revealed the ability of GLP-1(28–36) to shift mitochondrial substrate utilization from oxygen-consuming fatty acid metabolism towards oxygen-sparing glycolysis and glucose oxidation. Thus, the activation of sAC may be used as a preconditioning strategy to protect tissue against I/R injury. In contrast, Teodoro et al. [81] observed a protective effect against I/R injury in liver by pre-treatment with the sAC inhibitor LRE1. The authors found a significant improvement in respiration, ATP synthesis, and resistance to Ca^2+^ stress in mitochondria isolated after in vivo hepatic I/R in rats pre-treated with LRE1. The authors suggest that LRE1 pre-treatment leads to a mitohormetic response that protects mitochondrial function during I/R injury.

### 3.3. Heart Failure

Similar to our study [76], results published by Wang et al. [29] revealed that stimulation of endogenous sAC with HCO_3_^−^ as well as overexpression of sAC prevented the death of neonatal cardiomyocytes induced by camptothecin, H_2_O_2_ or TNF-α+actinomycin D. In contrast, inhibition of sAC with 2-hydroxyestradiol significantly aggravated cell death. The authors further applied an in vivo pathological model in which heart failure was induced in rats by transverse aortic constriction for 22 weeks. The study showed that constriction led to significant reduction of sAC expression, observed either in full tissue lysate or in the mitochondrial fraction, which was accompanied by reduced resistance of isolated mitochondria to Ca^2+^ stress, similar to the effect observed in control cardiac mitochondria after pharmacological sAC inhibition. The authors argue that at least some pathologies may lead to the downregulation of sAC expression, which in turn may have a detrimental effect on mitochondrial biology and cell fate.

### 3.4. Septic Cardiomyopathy

Septic cardiomyopathy, one of the key complications in clinical sepsis, is associated with adverse outcomes and increased mortality [82]. Dysregulated Ca^2+^ dynamics and mitochondrial dysfunction are among the key internal factors responsible for septic cardiomyopathy [83,84,85]. Numerous studies have demonstrated abnormalities in mitochondrial structure [86,87], oxidative stress [88,89], mitochondrial dynamics [90,91], and the quality control system [92]. Since these mitochondrial phenomena are regulated by mitochondrial sAC-generated cAMP, one can hypothesize that dysregulation of mitochondrial sAC-cAMP signalling occurs in sepsis. Indeed, mitochondrial PKA-mediated Ser58 phosphorylation of cytochrome c oxidase subunit IV-1, an essential regulatory mechanism for efficient oxidative metabolism [30], was reduced in septic hearts in vivo in a mouse model of sepsis [93]. However, a direct role of the mitochondrial sAC-cAMP signalling still needs to be verified. As described above, PDE2A is considered to be the predominant mitochondrial phosphodiesterase locally regulating cAMP levels in mitochondria [38]. Inhibition of PDE2A improved mitochondrial respiration in septic cardiac fibres in vitro but had limited impact on myocardial function in septic mice [93]. This suggests that cAMP levels generated by local inhibition of PDE2A may not be sufficient to activate mitochondrial OXPHOS to rescue sepsis-induced mitochondrial dysfunction, implying that additional mechanisms such as direct activation of sAC may be needed to generate sufficient levels of cAMP.

### 3.5. Amnesia

Exogenous cannabinoids induce amnesia, i.e., a deficit in memory. A recent report [36] showed a previously unknown link between acute brain mitochondrial activity and memory in which direct bioenergetic effects of cannabinoids play a central role in a sAC-dependent manner. The authors demonstrated that the type-1 cannabinoid receptors (mtCB1) are localized at the mitochondria. The activation of these receptors by cannabinoids leads to inhibition of sAC and, therefore, reduction of the cAMP level and PKA activity in mitochondria. This leads to diminished PKA-dependent phosphorylation/activation of OXPHOS proteins particularly complex I subunit NDUFS2. This cascade of events leads to a decrease in brain mitochondrial respiration and cellular ATP content, reduced mitochondrial mobility, synaptic depression, and eventually amnesia. In agreement, a recent study by Didier et al. [94] suggested the involvement of sAC, rather than tmAC, in inducing the expression of immediate early genes, a process responsible for the long-term memory formation, after glutamatergic synaptic activation by neurons.

### 3.6. Cancer

Accumulating evidence suggests a role of the sAC/cAMP pathway in the development and progression of various cancers. Depending on the type, it can either stimulate or inhibit the development of cancers. In prostate cancers, the expression of sAC is upregulated [95] and inhibition or knockdown of sAC suppresses the growth and enhances radiosensitivity of prostate cancer cells [95,96]. In contrast, its expression is downregulated in numerous human cancers, including bladder, skin, and renal cell carcinoma [97]. Moreover, in mouse embryonic fibroblasts (MEFs), it seems to act as an inhibitor of fibroblast transformation to cancer cells. Implantation of sAC knockout MEFs overexpressing SV40 in mouse results in development of larger tumours compared with those developing from wild-type MEFs [97].

However, the mechanisms of tumour promotion or suppression by sAC are largely unknown. One possible mechanism may be the modulation of cell metabolism. Cells meet their energy needs by glycolysis or by substrate oxidation via mitochondrial OXPHOS. Multiple signalling pathways precisely regulate both processes, and emerging data have revealed the critical role of sAC-generated cAMP under physiological and pathological conditions [16,24,27,92]. There is a tight balance between these processes, and both work in a coordinated manner to meet the energy demands of the cells. In several cancer types, however, this balance is shifted towards anaerobic glycolysis even in the presence of abundant oxygen, a phenomenon known as the Warburg effect [98]. Interestingly, in a study by Xing et al., the addition of a cell-permeable cAMP analogue as well as pharmacological activation of tmAC with forskolin reversed the Warburg effect in a glioblastoma cell line and inhibited tumour growth [99]. These cAMP effects were mediated via PGC-1α-dependant induction of mitochondrial biogenesis and improved mitochondrial function. In line with this, Chang and colleagues [100] recently showed that transient pharmacological suppression of sAC activity in various primary and cancer cell lines results in inhibition of OXPHOS and a metabolic shift towards glycolysis. This metabolic shift seems to be due to reduced activity of complex I resulting from the mitochondrial sAC inactivation. The authors propose these effects are mediated via inhibition of cAMP/EPAC signalling, as pharmacological inhibition of EPAC simulated the effects of sAC inhibition. Surprisingly, the authors observed increased glycogenolysis and upregulated anaerobic glycolysis by stimulation of tmAC with forskolin, which is opposite to the observations reported by Xing et al. [99]. Moreover, this observation conflicts with authors’ previous report [101] in which they demonstrated that bicarbonate-mediated activation of sAC in astrocytes induces glycolysis. The discrepancies between the studies by Xing et al. and Chang et al. may be due to differences in the duration of treatment with cAMP analogue. Chang et al. made the observations only after transient treatment, whereas in the study by Xing et al., cells were treated with cAMP analogue for 48 h. Table 1 gives a brief overview of pathologies associated with dysregulation of mitochondrial sAC-signalling.

## 4. Conclusions and Perspectives

Dysregulation of mitochondrial homeostasis may lead to severe diseases like cancer, neurodegenerative and cardiovascular diseases, diabetes, and inflammation. Numerous extra- and intramitochondrial signalling pathways provide essential transcriptional or post-translational control of mitochondrial function. Interestingly, mitochondria have several endogenous signalling pathways, separate from cytosolic pathways, that regulate post-translational mitochondrial protein modification and stability, particularly intra-mitochondrial cAMP signalling. The intramitochondrial cAMP pool is specifically generated by the matrix-localized sAC and significantly contributes to mitochondrial function, e.g., by the supporting activity of complexes I and IV at the mitochondrial electron transport chain. In addition, sAC-derived cAMP compartments are present at the OMM or IMS that may affect mitochondrial dynamics, mitophagy, and apoptosis. Therefore, disturbance of the sAC-dependent cAMP pool may impair mitochondrial homeostasis, contributing to various diseases. Indeed, emerging evidence suggests an association of the reduced sAC expression or activity with pathologies like heart failure [29], cancer [97], or cannabinoid-induced amnesia [36]. Mitochondrial dysfunction seems to be a causal link between the downregulation of sAC signalling and disease. These pioneer studies have opened a new avenue in biomarker discovery and therapy of a number of pathologies. Particularly, a clear understanding of the underlying cellular mechanisms leading to the sAC downregulation under some pathologies, e.g., cardiomyopathy [29], is required for the development of treatment strategies preventing the disturbance of the sAC-derived cAMP pool.

## Figures and Tables

**Table 1:** A brief overview of pathologies associated with dysregulation of mitochondrial sAC-signalling.

Pathologies	Species	sAC Alterations	Mitochondrial Effects	References
Amnesia	mice	Inhibition of activity	Reduced respiration	[36]
Ischemia/reperfusion	rats	Not studied	Mitochondrial apoptosis	[69,70,76]
Heart failure	rats	Reduced expression	Reduced mitochondrial Ca^2+^-resistance	[29]
Septic cardiomyopathy	mice	Not studied	Impaired ADP-stimulated respiratory rate	[93]
Prostate cancer	human	Upregulated	Not studied	[95,97]
Anaplastic oligoastrocytoma	human	Upregulated	Not studied	[97]
Several haematologic cancers	human	Downregulated	Not studied	[97]
Several solid cancers	human	Downregulated	Not studied	[97]
